# Supplementary material for: Leucine repeat adaptor protein 1 interacts with Dishevelled to regulate gastrulation cell movements in zebrafish
Source: Nat Commun. 2017 Nov 7;8:1353. doi: 10.1038/s41467-017-01552-x (PMC5677176; doi:10.1038/s41467-017-01552-x)
Supplement: Supplementary file 1 — Supplementary Information [file 41467_2017_1552_MOESM1_ESM.pdf]

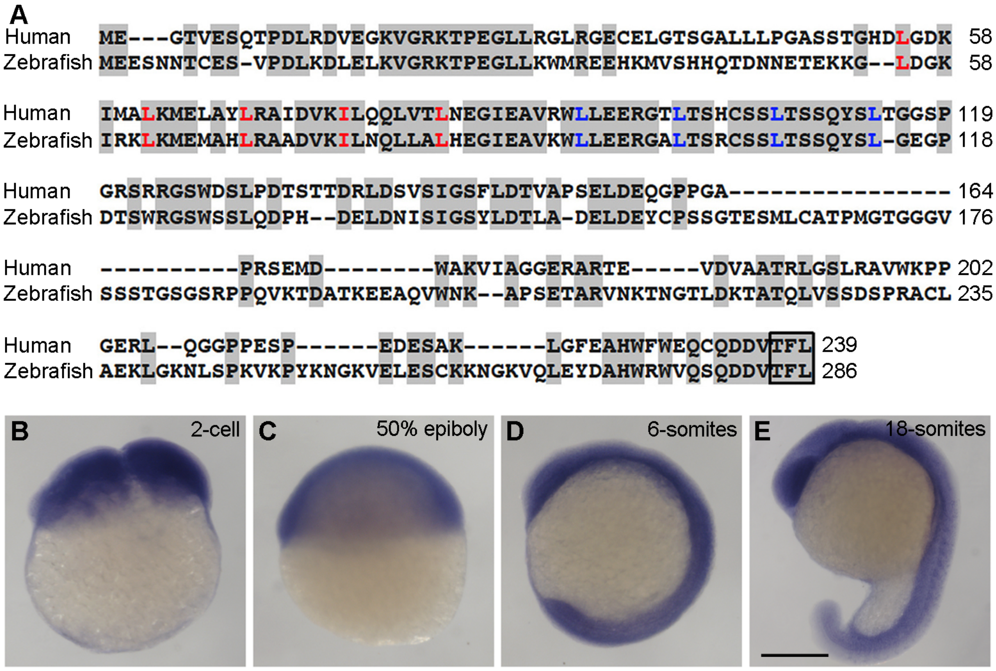

Supplementary Figure 1 | (**A**) Alignment of human and zebrafish Lurap1 protein sequences. Conserved residues are shadowed. The leucine (L) and isoleucine (I) residues in the tandem leucine repeats are indicated in red and blue, respectively. The C-terminus PDZ-binding motif is boxed. The numbers on the right indicate amino acid positions. (**B-E**) In situ hybridisation analysis of *lurap1* expression pattern in zebrafish embryos at indicated stages. Scale bar: (**B-E**) 200 μm.

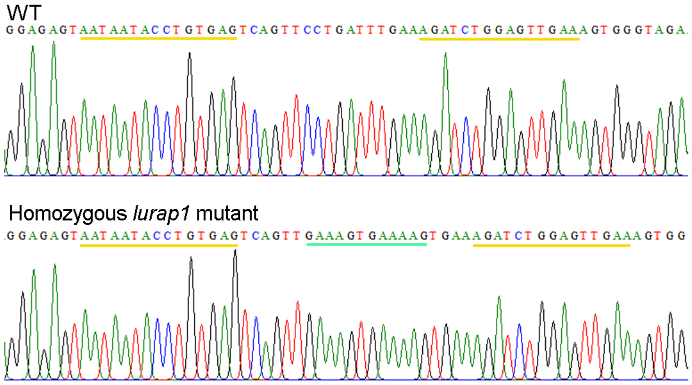

Supplementary Figure 2 | Sequencing chromatograms of the RT-PCR products amplified from WT embryos and homozygous *lurap1* mutants at 24 hpf. TALEN targeting sequences are underlined in yellow, and the insertion sequence is underlined in green.

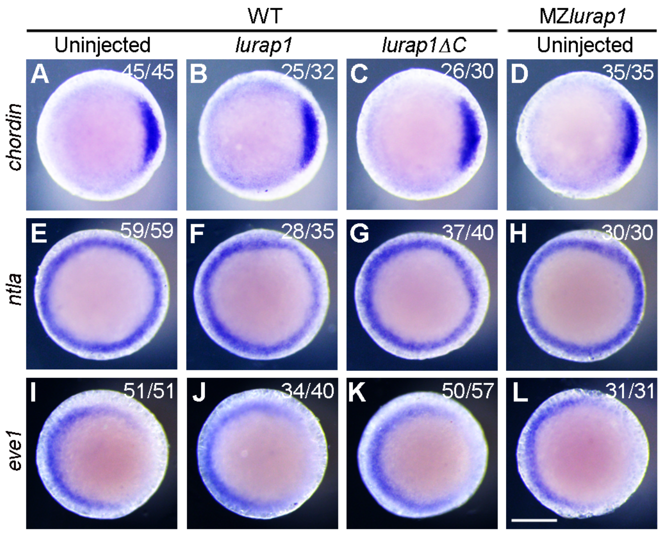

Supplementary Figure 3 | Early embryonic patterning is not affected in MZ*lurap1* embryos and in WT embryos overexpressing Lurap1 or Lurap1ΔC. The expression patterns of *chordin* (**A-D**), *ntla* (**E-H**), and *eve1* (**I-L**) were analysed in indicated embryos at shield stage. Animal pole view with dorsal on the right. Scale bar: 200 μm.

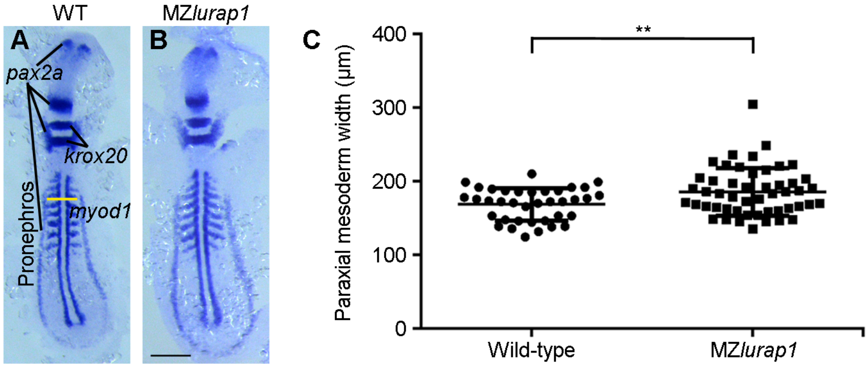

Supplementary Figure 4 | The defective CE phenotypes in MZ*lurap1* mutants are not caused by a developmental delay. (**A,B**) Simultaneous in situ hybridisation analysis of *pax2a*, *krox20*, and *myod1* expression patterns in flat-mounted WT and MZ*lurap1* embryos with equal somite number. The yellow horizontal line defines the inner sides of the pronephric anlages, and reflects the width of the paraxial mesoderm. (**C**) Scatter plot shows that MZ*lurap1* embryos display wider paraxial mesoderm. The paraxial mesoderm width was defined as the distance between the inner sides of the pronephric anlage at the third somite level. Bars represent the mean values ± s.d. from two independent experiments (**, P<0.01; Student's t-test). Scale bar: (**A,B**) 200 µm.

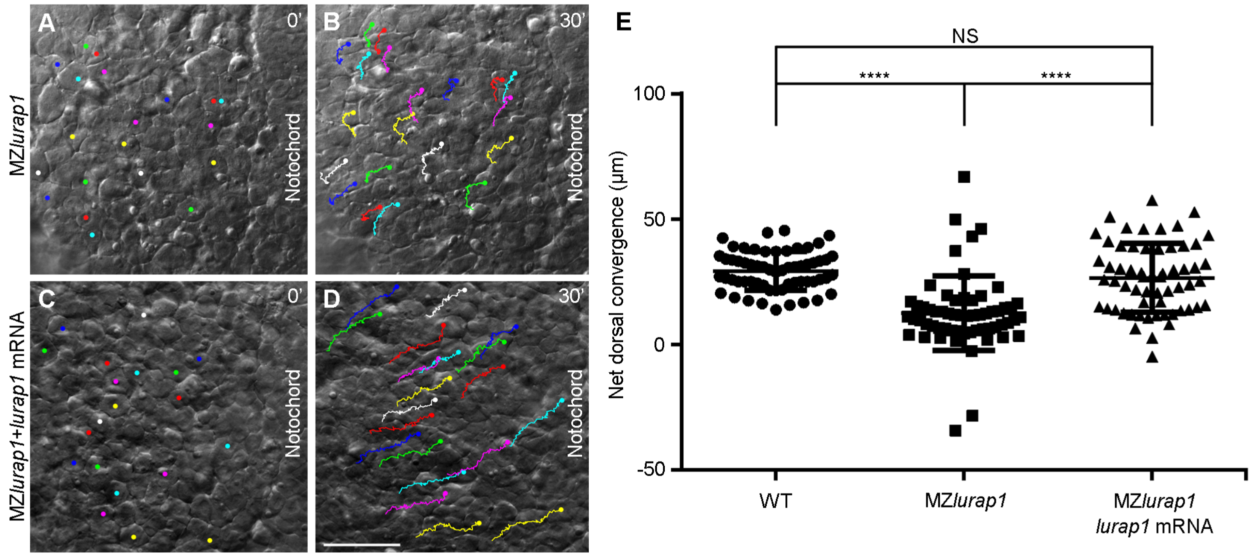

Supplementary Figure 5 | Lurap1 rescues dorsal convergence of lateral cells in MZ*lurap1* mutants. (**A-D**) Representative first and last images from live time-lapse movies show the convergence movement of lateral cells in uninjected MZ*lurap1*, and *lurap1*-injected MZ*lurap1* embryos, with the anterior region positioned on the top. (**E**) Scatter plot shows the net distance reached by lateral cells toward the notochord in indicated embryos. Bars represent the mean values ± s.d. from three independent embryos (****, $P<0.0001$; NS, not significant; Student's *t*-test). Scale bar: (**A-D**) 50 μm.

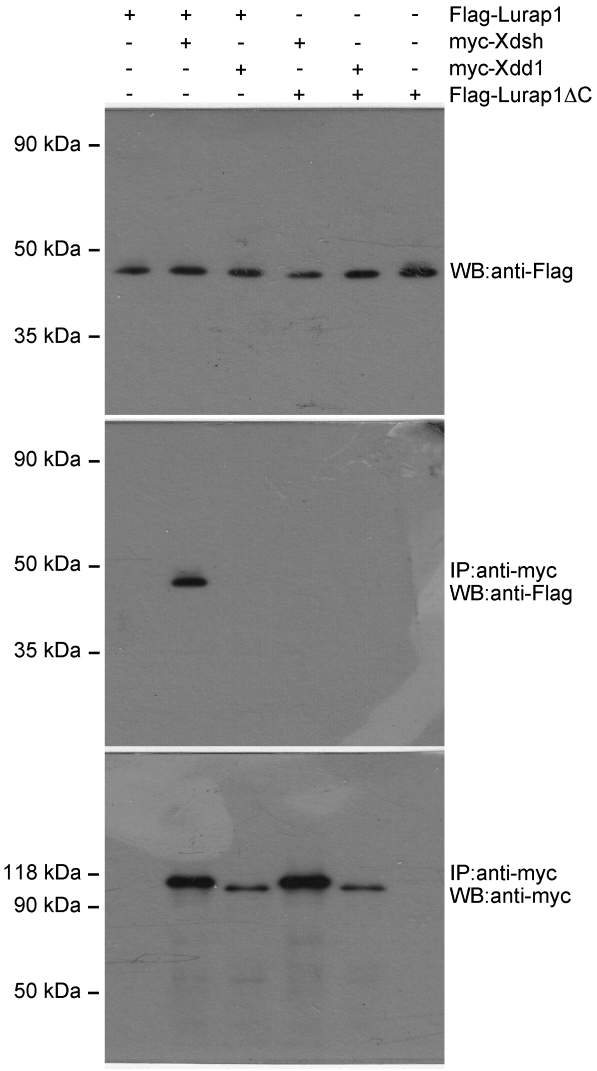

Supplementary Figure 6 | Physical interaction between Lurap1 and Dvl. Co-immunoprecipitation followed by western blotting. Uncropped scans of the whole western blots corresponding to Figure 4b in the main text.

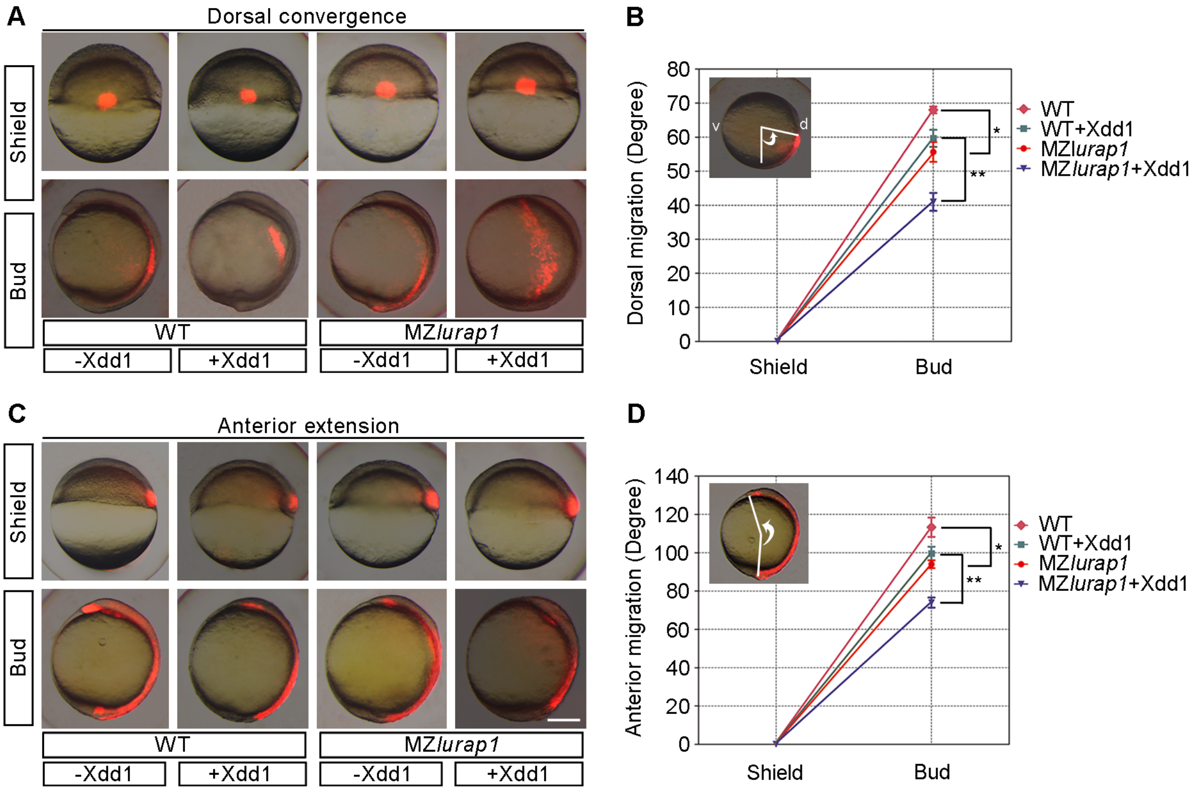

Supplementary Figure 7 | Functional interaction between Lurap1 and Xdd1 in CE movements. (**A**) Xdd1 enhances dorsal convergence defect in MZ*lurap1* embryos. (**B**) Statistical analysis of the convergence defect. The extent of convergence is reflected by the angle formed between the line connecting the centre of the embryo to the labelled cells, and the line perpendicular to the dorsal (d) and ventral (v) axis in animal pole view. Bars represent the mean values ± s.d. from three independent experiments using at least 30 embryos in each condition (*, P<0.05; **, P<0.01; Student's t-test). (**C**) Xdd1 enhances extension movement defect in MZ*lurap1* embryos. (**D**) Statistical analysis of the extension defect. The extent of extension is represented by the angle between the most anteriorly and the most posteriorly labelled cells, with vertex at geometric centre of the embryo. Bars represent the mean values ± s.d. from three independent experiments using at least 30 embryos in each condition (*, P<0.05; **, P<0.01; Student's t-test). Scale bar: (**A,C**) 200 μm.

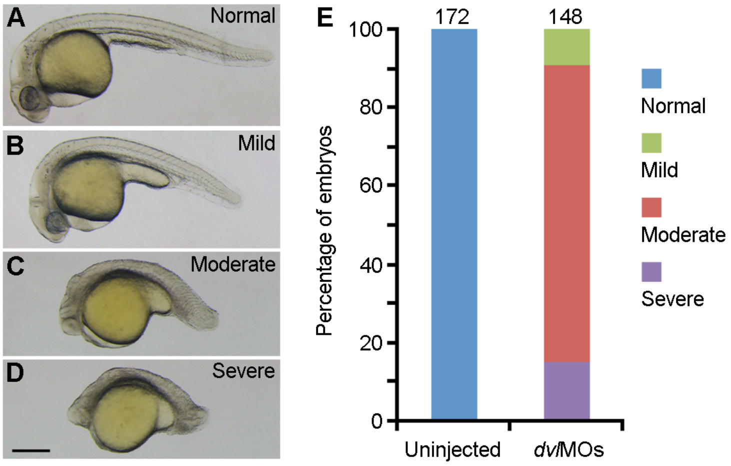

Supplementary Figure 8 | Defective CE phenotypes in *dvl2* and *dvl3a* double morphants. (**A-D**) The embryos were injected with 4 ng *dvl2*MO and 10 ng *dvl3a*MO (*dvl*MOs), and the resulting phenotypes were observed at 24 hpf and grouped into 4 categories, as indicated. (**E**) Graph shows the percentage of different phenotypes scored from three experiments, with total number of embryos shown on the top of each stacked column. Scale bar: (**A-D**) 200 μm.

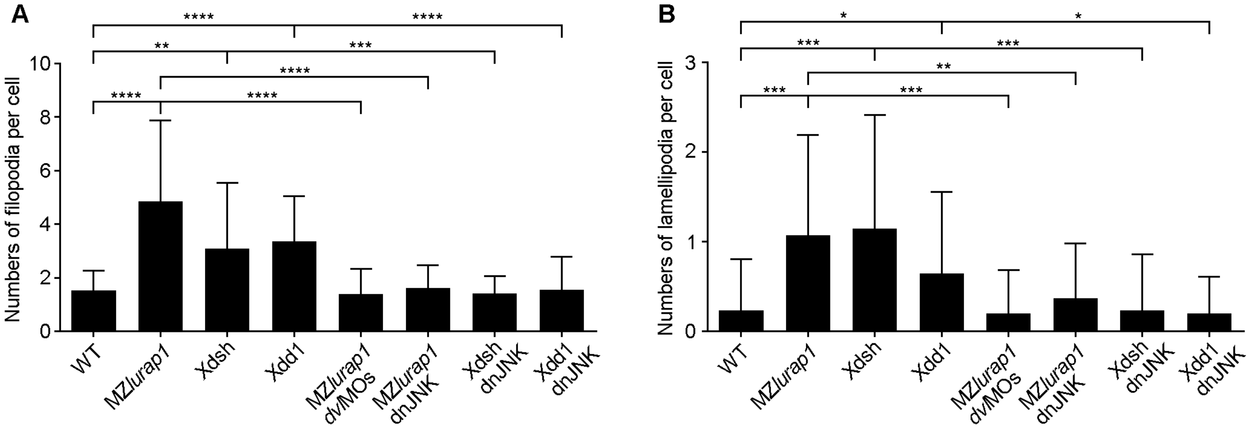

Supplementary Figure 9 | Analyses of the type and orientation of cellular protrusions. (**A**) Comparison of filopodia in indicated conditions. (**B**) Comparison of lamellipodia in indicated conditions. Bars represent the mean values ± s.d. from three independent experiments by examining a total of 30 cells derived from 8 to 10 embryos in each condition (*, P<0.05; **, P<0.01; ***, P<0.001; ****, P<0.0001; Student's t-test). Xdsh-overexpressing cells form more proportion of lamellipodia than Xdd1-overexpressing cells.

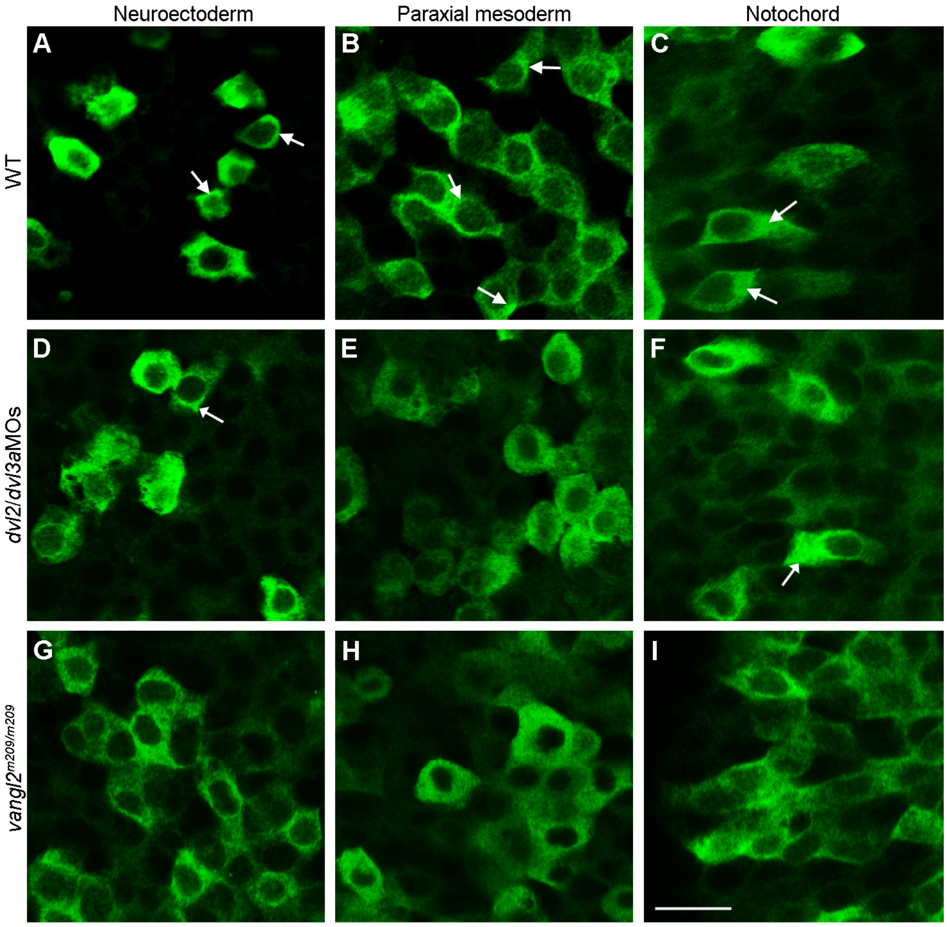

Supplementary Figure 10 | Analysis by confocal microscopy of the subcellular localisation of myc-tagged Lurap1 in different germ layers at 80% epiboly stage, as indicated. (**A-C**) Subcellular localisation of Lurap1 in a WT embryo. Some cells (arrows) show localised distribution. (**D-F**) Subcellular localisation of Lurap1 in a *dvl2* and *dvl3a* morphant embryo. (**G-I**) Subcellular localisation of Lurap1 in a zygotic *vangl2*$^{m209/m209}$ mutant. Scale bar: (**A-I**) 20 μm.

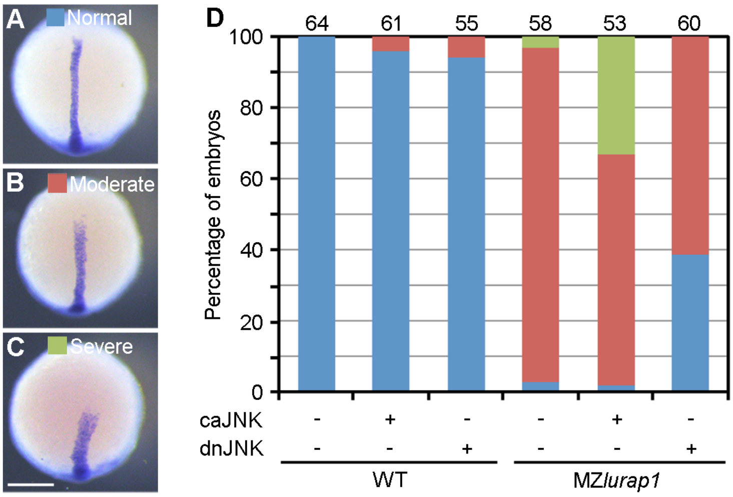

Supplementary Figure 11 | Functional interaction between Lurap1 and JNK signalling in CE movements. (**A-C**) Analysis of *ntla* expression pattern to assess the extent of CE defects. All embryos were analysed at the bud stage and classified into three groups, as indicated. (**D**) The defective CE phenotypes in MZ*lurap1* embryos are enhanced by caJNK, and rescued by dnJNK. The data were obtained from three experiments, with total number of embryos shown on the top of each stacked column. Scale bar: (**A-C**) 200 μm.
