# Supplementary material for: Leucine repeat adaptor protein 1 interacts with Dishevelled to regulate gastrulation cell movements in zebrafish
Source: Nat Commun. 2017 Nov 7;8:1353. doi: 10.1038/s41467-017-01552-x (PMC5677176; doi:10.1038/s41467-017-01552-x)
Supplement: Supplementary file 3 — Description of Additional Supplementary Files [file 41467_2017_1552_MOESM3_ESM.docx]

Description of Additional Supplementary Files

File Name: Supplementary Movie 1

Description: Dorsal convergence of lateral cells in a wild-type embryo. The anterior region is oriented to the top, and the notochord is on the right side. Coloured dots represent 20 randomly selected cells to follow their movements.

File Name: Supplementary Movie 2

Description: Dorsal convergence of lateral cells in an MZ*lurap1* mutant. The anterior region is oriented to the top, and the notochord is on the right side. Coloured dots represent 20 randomly selected cells to follow their movements.

File Name: Supplementary Movie 3

Description: Anterior extension of notochord cells in a wild-type embryo. The anterior region is oriented to the top, with coloured dots representing 20 randomly selected cells.

File Name: Supplementary Movie 4

Description: Anterior extension of notochord cells in an MZ*lurap1* mutant. The anterior region is oriented to the top, with coloured dots representing 20 randomly selected cells.

File Name: Supplementary Movie 5

Description: Rescue of dorsal convergence in an MZ*lurap1* mutant by Lurap1. To be compared with Supplementary Movie 2.

File Name: Supplementary Movie 6

Description: Rescue of anterior extension in an MZ*lurap1* mutant by Lurap1. To be compared with Supplementary Movie 4.

File Name: Supplementary Movie 7

Description: Bipolar behaviours of a wild-type notochord cell. The cell membrane is labelled with mGFP, and the nucleus is labelled with Histone2B-RFP. The cell is elongated and form essentially filopodia in mediolateral direction. Anterior region is oriented on the top.

File Name: Supplementary Movie 8

Description: Multipolar behaviours of MZ*lurap1* notochord cells. The cells are not elongated, and they develop multiple long filopodia in randomised directions. Anterior region is oriented on the top.

File Name: Supplementary Movie 9

Description: Randomised blebs in MZ*lurap1* neuroectoderm cells. The cells are round in shape, and form random blebs in all directions. Anterior region is oriented on the top.

File Name: Supplementary Movie 10

Description: Multipolar behaviours of an Xdsh-overexpressing notochord cell. The cell is not elongated, and form multiple lamellipodia and filopodia in all directions. Anterior region is oriented on the top.

File Name: Supplementary Movie 11

Description: Multipolar behaviours of an Xdd1-overexpressing notochord cell. The round-shaped cell extends multiple filopodia in all directions. Anterior region is oriented on the top.

File Name: Supplementary Movie 12

Description: Knockdown of *dvl2* and *dvl3a* disrupts notochord cell polarity. The cell is round in shape, and shows absence of cellular protrusion. Anterior region is oriented on the top.

File Name: Supplementary Movie 13

Description: Multipolar behaviours of caJNK-overexpressing notochord cells. The cells are not elongated and develop multiple long filopodia in randomised directions. Anterior region is oriented on the top.

File Name: Supplementary Movie 14

Description: Lack of cellular protrusion in dnJNK-overexpressing notochord cells. The cells are round in shape, and show absence of cellular protrusion. Anterior region is oriented on the top.

File Name: Supplementary Movie 15

Description: Rescue of cell polarity in an MZ*lurap1* mutant notochord cell by reducing Dvl dosage. Low amounts of *dvl2*MO and *dvl3a*MO rescues mediolateral polarity and bipolar activity. Anterior region is oriented on the top.

File Name: Supplementary Movie 16

Description: Rescue of cell polarity in an MZ*lurap1* mutant notochord cell by dnJNK. Inhibition of JNK activity rescues mediolateral polarity and bipolar activity. Anterior region is oriented on the top.

File Name: Supplementary Movie 17

Description: Rescue of cell polarity in Xdsh-overexpressing notochord cells by dnJNK. Inhibition of JNK activity rescues mediolateral polarity and bipolar activity. Anterior region is oriented on the top.

File Name: Supplementary Movie 18

Description: Rescue of cell polarity in an Xdd1-oxerexpressing notochord cell by dnJNK. Inhibition of JNK activity rescues mediolateral polarity and bipolar activity. Anterior region is oriented on the top.

File Name: Supplementary Movie 19

Description: MTOC positioning in a wild-type cell. The centriole is labelled with centrin4-GFP, and the cell membrane and nucleus are labelled with mRFP and Histone2B-RFP, respectively. The centriole moves essentially at the posterior position of the nucleus, as traced by the blue line. Anterior region is oriented on the top.

File Name: Supplementary Movie 20

Description: MTOC positioning in an MZ*lurap1* mutant cell. The centriole constantly changes its anteroposterior positions with respect to the nucleus. Anterior region is oriented on the top.

File Name: Supplementary Movie 21

Description: MTOC positioning in a caJNK-overexpressing cell. The centriole constantly changes its anteroposterior positions with respect to the nucleus. Anterior region is oriented on the top.
